# Assessing the Impact of Mesoporous, Co-Amorphous, and Polymer-Based Systems on Cefdinir’s Dissolution and Stability Via Predictive Modeling

**DOI:** 10.3389/bjbs.2026.15242

**Published:** 2026-02-13

**Authors:** Raghad Al Nuss, Mohamad Anas Al Tahan, Hind El-Zein

**Affiliations:** 1 Department of Pharmaceutics and Pharmaceutical Technology, Faculty of Pharmacy, Arab International University, Daraa, Syria; 2 Aston Medical School, College of Health and Life Sciences, Aston University, Birmingham, United Kingdom; 3 Department of Pharmaceutics and Pharmaceutical Technology, Faculty of Pharmacy, Damascus University, Damascus, Syria

**Keywords:** cefdinir, co-amorphous systems, dissolution enhancement, drug stability, mesoporous silica

## Abstract

The poor solubility and permeability of Biopharmaceutics Classification System (BCS) Class IV drugs pose major challenges to achieving sufficient oral bioavailability and therapeutic efficacy. Improving drug dissolution is a key strategy to enhance bioavailability, which in turn can enable more effective targeting of drugs to their site of action. To address this, we formulated cefdinir, a model BCS Class IV compound, using three amorphisation strategies; solid dispersions, mesoporous silica dispersions, and co-amorphous systems to assess the impact of formulation on stability and dissolution. Formulations were prepared via spray drying and solvent immersion using different drug-to-polymer ratios, with miscibility predicted using Flory–Huggins theory. The amorphous nature of each system was confirmed using differential scanning calorimetry (DSC), polarised light microscopy (PLM), and powder X-ray diffraction (PXRD). Dissolution studies revealed significantly enhanced drug release from all formulations compared to crystalline cefdinir. Among them, solid dispersion and co-amorphous systems exhibited the greatest improvement in dissolution rates, attributed to their ability to maintain supersaturation and inhibit crystallisation via kinetic stabilisation. These systems also showed better physical stability under non-sink aqueous conditions. However, mesoporous silica dispersions demonstrated superior long-term stability, retaining over 95% drug content and preserving their amorphous structure across three storage conditions (25 °C/0% RH, 40 °C/0% RH, and 40 °C/75% RH) for 6 months. This was attributed to the confinement of the drug within silica pores and the absence of hygroscopic excipients. Overall, this study highlights the distinct advantages of each approach, emphasising the importance of balancing dissolution enhancement with solid-state stability, and supports the use of theoretical modelling to guide rational formulation design for poorly soluble drugs to improve oral bioavailability and enable more targeted therapeutic outcomes.

## Introduction

Poor aqueous solubility remains one of the major challenges in pharmaceutical development, particularly for many newly discovered active pharmaceutical ingredients (APIs) [1]. The rise of combinatorial chemistry in drug discovery has contributed to a proliferation of lipophilic compounds that are poorly soluble in water, often resulting in limited bioavailability [2]. According to the Biopharmaceutics Classification System (BCS), drugs are categorised into four classes based on their water solubility and membrane permeability. BCS Class II compounds are poorly soluble but highly permeable, while Class IV drugs suffer from both poor solubility and low permeability, making them the most difficult to formulate effectively [3]. Numerous strategies have been developed to enhance the solubility of such compounds, including micronisation [4–6], nanosuspensions [7–9], and cyclodextrin complexation [10–12]. Among these, amorphization, transforming a crystalline drug into its amorphous form remains one of the most promising approaches [13, 14]. The amorphous state lacks long-range molecular order and possesses higher internal energy, leading to improved apparent solubility and faster dissolution rates. This can further affects their interaction with cells and tissues as improving the physiochemical properties enhances their cellular targeting characteristics [15] However, this form is thermodynamically unstable and prone to recrystallisation during manufacturing, storage, or even *in vivo*, which may negate its solubility advantages [16, 17].

To overcome the inherent instability of amorphous drugs, various glass solution systems have been developed using stabilising excipients. The most widely studied approaches include polymer-based amorphous solid dispersions (ASDs), mesoporous silica-based systems, and co-amorphous formulations, each designed to inhibit recrystallisation and enhance the physical stability of the amorphous form [18].

ASDs involve dispersing one or more APIs at the molecular level within hydrophilic polymer matrices. Stabilisation is achieved through mechanisms such as kinetic hindrance of crystallisation, elevation of the glass transition temperature (Tg), specific drug–polymer interactions (e.g., hydrogen bonding), and physical separation through molecular-level mixing [18, 19] Mesoporous silica systems, including materials such as SBA-15 (used in this study), colloidal silicon dioxide, Neusilin®, and Florite®, stabilise amorphous drugs through nanoconfinement within pores ranging from 2 to 50 nm. This confinement reduces molecular mobility and prevents recrystallisation. Additionally, surface silanol groups interact with drug molecules, further enhancing stability [18, 20, 21]. Furthermore, the use of silica-based systems can prove helpful for targeting applications, whether towards cancerous cells, colon targeting, or magnetic targeting [22]. Co-amorphous systems, in contrast, employ low molecular weight co-formers such as amino acids, sugars, or urea to stabilise the amorphous phase. These co-formers may act via intermolecular interactions, Tg enhancement, or uniform molecular dispersion. Amino acids are particularly favoured for their dual role in dissolution enhancement and physical stabilisation [18, 23, 24].

Across all these systems, the careful selection of a compatible stabilising agent is critical to success. To aid in this, several theoretical models have been proposed, including the solubility parameter approach [25], Flory–Huggins interaction theory [26], melting enthalpy-based miscibility predictions [27], and molecular simulations [28]. These tools can provide valuable insights during formulation design by predicting excipient–drug compatibility and helping to optimise stability profiles.

The aim of this study is to comparatively evaluate the effectiveness of three stabilisation strategies: amorphous solid dispersions, mesoporous silica systems, and co-amorphous formulations in maintaining the amorphous structure and ensuring stability in both aqueous environments and the solid state. Cefdinir, a third-generation broad-spectrum cephalosporin antibiotic and a representative BCS Class IV drug, was selected as the model compound due to its poor solubility and limited permeability [29], which contribute to its low oral bioavailability, reported to range between 21% and 25% [30]. To support rational formulation design, the Hansen solubility parameter approach and Flory–Huggins theory were employed to predict drug–excipient miscibility, thereby enabling the selection of one optimised formulation from each stabilisation approach. This theoretical framework allowed for a focused comparison of their performance.

The selected formulations were then subjected to a range of stability conditions, including accelerated stability testing (40 °C/75% RH), dry storage (25 °C/0% RH and 40 °C/0% RH), and aqueous incubation, in order to assess their resistance to recrystallisation and chemical degradation over time. While each method offers its own advantages, to our knowledge no prior study has systematically compared these three strategies across multiple performance dimensions, namely, dissolution enhancement, physical stability, chemical stability, and thermal resistance. This study aims to address this gap and identify the most robust stabilisation system for improving the delivery of poorly soluble and poorly permeable drugs such as cefdinir.

## Materials and Methods

### Materials

Cefdinir was supplied by Lupin Co. Ltd. (India). Polyvinylpyrrolidone K30 (PVP K30), hydroxypropyl methylcellulose (HPMC 606), and Eudragit L100 were sourced from Ashland Inc. (USA), Shin-Etsu (Japan), and Evonik Industries (Germany), respectively. SBA-15 mesoporous silica was purchased from Jiangsu XFNANO (China). L-arginine, L-tryptophan and L-phenylalanine were obtained from Titan Biotech LTD (India). All solvents and reagents were of analytical grade.

### Methods

#### Theoretical Miscibility Prediction

Theoretical miscibility predictions were conducted for a range of stabilisers to guide the selection of optimal candidates for each formulation strategy. These included polymers: PVP K30, HPMC 606, and Eudragit L100, for amorphous solid dispersions; SBA-15 as the mesoporous silica carrier; and amino acids: L-arginine, L-tryptophan, and L-phenylalanine for co-amorphous systems. This predictive screening enabled the rational selection of the most suitable stabiliser within each approach, thereby facilitating a robust and systematic comparison across the three amorphisation strategies.

##### Hansen Solubility Parameter Estimation

The Hansen Solubility Parameters (HSP) for cefdinir and all excipients were calculated using the group contribution method to assess drug–excipient miscibility. To determine the interaction radius (Ra) according the following equation, three components were evaluated: dispersion (δd), polarity (δp), and hydrogen bonding (δh). Ra was calculated according to the following equation [31]:

$$\sqrt{\text{Ra}={4\left({\delta d}^{1}-{\delta d}^{2}\right)}^{2\;}+{\left({\delta p}^{1}-{\delta p}^{2}\right)}^{2\;}+{\left({\delta h}^{1}-{\delta h}^{2}\right)}^{2\;}\;}$$

Smaller Ra values indicate improved miscibility. The results informed excipient selection and corroborated observed stabilisation trends. It was found that systems with values below 7 MPa½ are miscible, whereas those with values above 10 MPa½ are likely to be immiscible [25].

##### Flory–Huggins Interaction Parameter Calculation

To further investigate the miscibility between cefdinir and each excipient, Flory–Huggins theory was employed for thermodynamic predictions of miscibility. This approach, based on the difference in solubility parameters and molar volume, allowed the calculation of the interaction parameter (χ) using the following equation [25]:

$$X=\frac{Vm}{RT}\;{\left(\;\delta \text{drug}-\delta \text{excepient}\right)}^{2}$$

Where Vm is the molar volume of the drug (cm^3^/mol), R is the gas constant (8.314 J/mol·K), T is the absolute temperature (298 K), and *δ* denotes the total solubility parameter of the drug and excipient, respectively, expressed in MPa½. The total solubility parameters (δt) were calculated from the previously determined Hansen components using the following relation [31]:

$$\delta t=\sqrt{{\delta d}^{2}+{\delta p}^{2}+{\delta h}^{2}}$$

The molar volume of cefdinir was estimated using the group contribution method. χ values were calculated for PVP K30, HPMC 606, Eudragit L100, SBA-15, and the amino acid coformers (L-arginine, L-phenylalanine, and L-tryptophan), and interpreted according to standard miscibility thresholds, where χ < 0.5 indicates good miscibility [25].

#### Formulation Preparation

##### Polymeric Amorphous Solid Dispersion (Selected: PVP K30-Based)

Amorphous solid dispersions of cefdinir were prepared via spray-drying, using PVP K30 as the polymeric carrier at a 1:2 (drug-to-polymer) weight ratio. This ratio was selected based on miscibility calculations and our previous study, which demonstrated enhanced dissolution performance across three biorelevant media (pH 1.2, 4.5, and 6.8) [32]. The drug and polymer were dissolved in deionised water adjusted to approximately pH 7.0 using 1 N sodium hydroxide to ensure complete solubilisation of the drug. The solution was then spray-dried using a Büchi B-191 Mini Spray Dryer (Germany) under the following conditions: inlet temperature 160 °C, outlet temperature 83 °C ± 6 °C, aspirator setting 100%, feed rate 5.3 ± 0.2 mL/min, and airflow rate 600 L/h. The resulting amorphous solid dispersions were collected, sealed in amber glass vials, and stored in a desiccator until further analysis.

##### Preparation of Mesoporous Silica-Based System (Solvent Immersion Method)

Cefdinir was loaded into mesoporous silica (SBA-15) using the solvent immersion method. Based on our previous study, solubility screening identified *n*-hexane as the most effective solvent for drug loading, achieving a maximum efficiency of 37% w:w [33]. Accordingly, a cefdinir solution in *n*-hexane was prepared at a concentration of 30 mg/mL and added to SBA-15 (25 mg) at a fixed silica-to-solution ratio of 1:1000 (w/v). The mixture was stirred continuously for 24 h at room temperature (25 °C) to facilitate adsorption of the drug into the mesoporous matrix. Following incubation, the loaded silica was separated by centrifugation at 8000 rpm for 30 min. The solid was then air-dried under ambient conditions for 72 h, followed by oven-drying at 60 °C until a constant weight was achieved. The final cefdinir–SBA-15 formulation was stored in sealed glass vials under dry conditions until further analysis.

##### Preparation of Co-Amorphous Formulation

Co-amorphous formulations of cefdinir were prepared using two amino acid coformers, L-arginine and L-phenylalanine, at a 1:1:1 M ratio. The components were dissolved in distilled water to produce clear solutions, avoiding the use of organic solvents. The resulting aqueous solution was spray-dried using a Büchi B-191 Mini Spray Dryer (Germany) under the following conditions: inlet temperature 160 °C, outlet temperature 83 °C ± 6 °C, aspirator setting 100%, feed rate 5.3 ± 0.2 mL/min, and airflow rate 600 L/h. The dried powders were collected in amber glass vials and stored in a desiccator until further evaluation. The detailed composition of the three prepared formulations is presented in Table 1.

**TABLE 1:** Compositions of the different prepared formulations.

Formulation	F1 (polymeric solid dispersion)	F2 (mesoporous silica-based system)	F3 (co-amorphous system)
Cefdinir	300 mg	300 mg	300 mg
Pvp-K30	600 mg	_	_
Na OH	30.35 mg	_	_
SBA-15	_	507.2 mg	_
L- arginine	_	_	174.20 mg
L-phenylalanine	​	​	165.19 mg

#### Drug Content and Yield

The percentage yield of the prepared formulations was determined by comparing the final weight of the dried product to the total initial weight of the drug and excipients used, using the following formula [32]:

$$\text{Yield}=(\frac{\text{Actual}\;\text{weight}\;\text{of}\;\text{dried}\;\text{product}}{\text{Initial}\;\text{total}\;\text{weight}\;\text{of}\;\text{drug}+\text{excipient}})\times 100$$

Drug content was calculated as follows: a weighed amount of each formulation, equivalent to 50 mg of cefdinir, was dissolved in 50 mL of phosphate buffer (pH 7.0), sonicated, filtered through a 0.45 µm membrane filter, appropriately diluted, and analysed using the pharmacopeial HPLC method. Analysis was carried out on an HPLC apparatus (Jasco, Japan) equipped with a Knauer C18 column (4.6 × 150 mm, packing L1) and a UV-VIS detector (Model UV-970, Jasco, Japan). The mobile phase consisted of methanol, tetrahydrofuran, and citric acid monohydrate solution (111:28:1000, v/v), adjusted to pH 2.0 ± 0.05 using phosphoric acid. The flow rate was set at 1.4 mL/min, with an injection volume of 15 μL, and detection was carried out at 254 nm. The cefdinir content in each sample was quantified using the following equation [34]:

$$\text{Drug content }\left(\%\right)=\left(\frac{Sample\;peak\;area\;X\;Standard\;concentration}{Standard\;peak\;area\;X\;Nominal\;conventration}\right)\;X\;100$$

#### Polarised Light Microscopy (PLM)

Polarised light microscopy (PLM) was employed as a preliminary method to assess the crystallinity of the prepared formulations. Samples were dispersed onto glass slides, and PLM was carried out using a polarising microscope (Olympus BX41, USA) at ×40 magnification with crossed polarisers. Birefringence was interpreted as indicative of residual or recrystallised crystalline domains, whereas the absence of birefringence was considered evidence of an amorphous structure [35, 36].

#### Thermal Stability Study

Thermal analysis of the formulations was conducted using a differential scanning calorimeter (DSC 131; SETARAM, France) to assess thermal behaviour and stability. Approximately 5 mg of each sample was weighed and sealed in standard aluminium pans, with an empty sealed pan used as a reference. Thermal scans were run from 30 °C to 300 °C at a heating rate of 10 °C/min under a nitrogen purge (50 mL/min). Although the DSC could theoretically be started at lower temperatures, preliminary studies and literature precedent indicate that no relevant thermal transitions for cefdinir occur below 30 °C. For example, in a previous study, thermal scans for other materials were conducted from 25 °C without loss of relevant thermal events [21]. Thermograms were analysed for melting endotherms and glass transition temperature (Tg) events, providing insights into the amorphous nature and thermal stability of cefdinir in the formulations [37]. Each measurement was performed in triplicate to ensure reproducibility.

#### Powder X-Ray Diffraction (PXRD)

Pure cefdinir and all prepared formulations, including drug–excipient mixtures, were characterised using X-ray diffraction (XRD) with a Bruker D8 Advance diffractometer (West Germany), equipped with a germanium monochromator and a copper radiation source filtered through nitrogen. The instrument was operated at 50 kV and 30 mA. Diffraction patterns were recorded over a 2θ range of 5°–60° to assess the physical state of cefdinir within the formulations relative to the pure drug [37].

#### Dissolution Studies

The dissolution behaviour of cefdinir in the prepared formulations was evaluated using USP Apparatus II (paddle method) on a PT-DT7 dissolution tester (Pharma Test, Germany). Tests were conducted at 50 rpm and 37 °C ± 0.2 °C, using 900 mL of dissolution medium to simulate gastrointestinal conditions: HCl buffer (pH 1.2), acetate buffer (pH 4.5), and phosphate buffer (pH 6.8). These three media were selected to represent the varying pH environments of the human gastrointestinal tract, enabling assessment of pH-dependent solubility, dissolution kinetics, and formulation performance across physiologically relevant conditions. An amount equivalent to 300 mg of cefdinir was used per test. Aliquots (5 mL) were withdrawn at predetermined time intervals (2, 5, 10, 15, 20, 30, 45, and 60 min), filtered through a 0.45 µm syringe filter, and replaced with an equal volume of fresh medium to maintain sink conditions. The concentration of cefdinir released was determined using UV–VIS spectrophotometry (T80, PG Instruments, UK) at 280 nm, 286 nm, and 287 nm for HCl, acetate, and phosphate buffers, respectively. Each dissolution profile represents the mean of three replicates, and statistical comparisons were made between formulations [34]. Although saturated solubility measurements were not explicitly performed, the dissolution medium volumes were selected to be at least 3–5 times greater than the expected maximum solubility of cefdinir, and aliquot replacement was used to approximate sink conditions. This approach ensured that the formulations remained fully dissolved throughout the testing period.

#### Chemical Stability Studies

##### Chemical Stability

The chemical stability of cefdinir in the formulations was assessed by quantifying drug degradation under accelerated storage conditions. Samples collected at 3 and 6 months were analysed for cefdinir content using the validated HPLC method described earlier.

#### Physical Stability Study

##### Aqueous Solutions

The physical stability of amorphous cefdinir was assessed in selected formulations under non-sink conditions, designed to mimic physiological stress during administration. Samples containing 300 mg of cefdinir were suspended in 250 mL of distilled water at 37 °C ± 0.5 °C, using USP Apparatus II (paddle method) at 50 rpm. Aliquots (5 mL) were withdrawn at specific time intervals (2, 5, 10, 15, 20, 30, 45, 60, 120, and 180 min), filtered immediately through a 0.45 µm membrane filter, diluted, and analysed using a UV–VIS spectrophotometer (T80, PG Instruments, UK) at 287 nm. Each sample withdrawal was followed by replacement with an equal volume of distilled water. The data were interpreted in terms of the tendency of amorphous cefdinir to recrystallise during early dissolution in aqueous suspension.

##### Solid State

The physical stability of amorphous cefdinir in the selected formulations was further investigated under accelerated storage conditions at three environmental settings that included 25 °C/0% relative humidity (RH), 40 °C/0% RH, and 40 °C/75% RH. Zero humidity was maintained using phosphorus pentoxide (P_2_O_5_) in desiccators placed in ovens, while high humidity conditions (75% RH) were achieved using saturated sodium chloride (NaCl) solutions in desiccators kept at 40 °C. All samples were stored in the dark and analysed at predefined intervals: 1 week, 2 weeks, 1 month, 2 months, 3 months, and 6 months. PXRD was employed to monitor physical changes, specifically recrystallisation.

#### Statistical Analysis

Statistical analyses were performed using SPSS version 28 software. One-way analysis of variance (ANOVA) followed by Tukey’s *post hoc* test was used to assess differences between groups. All experiments were conducted in triplicate. Data are presented as mean ± standard deviation (SD), and a p-value <0.05 was considered statistically significant.

## Results

### Hansen Solubility Parameters (HSPs)

Table 2 presents the calculated Hansen Solubility Parameters (δ) and Ra values between cefdinir and each excipient. A lower Ra indicates stronger molecular affinity. Among the polymers, HPMC 606 and PVP K30 exhibited the strongest compatibility with cefdinir, while Eudragit L100 showed borderline compatibility, as reflected by its relatively higher Ra. SBA-15 displayed the highest Ra, indicative of poor miscibility, consistent with its expected role as a passive matrix rather than an interacting carrier. Of the amino acids evaluated, L-tryptophan and L-phenylalanine showed Ra values closest to cefdinir, suggesting favourable interactions, whereas L-arginine exhibited a higher Ra, reflecting less favourable solubility-based compatibility (Table 2). The Flory–Huggins interaction parameter (χ) values, summarised in Table 3, provide complementary insight into the thermodynamic miscibility between cefdinir and each excipient. A χ value <0.5 indicates thermodynamic compatibility [31]. All polymers displayed χ values below this threshold, with HPMC 606 showing the lowest χ (0.076), followed by PVP K30 and Eudragit L100, confirming favourable miscibility. Similarly, all amino acids demonstrated χ < 0.5, indicating good miscibility; notably, L-arginine had the lowest χ value (0.260), suggesting the strongest predicted thermodynamic compatibility among the amino acids tested.

**TABLE 2:** Results of Hansen solubility parameter calculations.

Material	δ_d_ (MPa^0.5^)	δ_p_ (MPa^0.5^)	δ_h_ (MPa^0.5^)	R_a_ vs. cefdinir (MPa^0.5^)
Cefdinir	17.29	8.2	11.2	​
Polymers				
PVP-K30	18.5	8.0	12.0	4.11
HPMC-606	17.4	10.2	10.9	3.93
Eudragit L100	17.6	9.5	6.9	5.32
Mesoporous silica				
SBA-15	15.2	3.0	8.0	7.92
Amino acids				
L- arginine	16.8	14.2	17.5	4.77
L- phenylalanine	18.3	8.4	11.2	3.25
L- tryptophan	17.9	10.5	12.4	2.94

**TABLE 3:** The calculated Flory–Huggins interaction parameters (χ) between cefdinir and all the studied stabilizing agents.

Cefdinir- excipient pair	χ value
Cefdinir–PVP K30	0.098
Cefdinir–HPMC 606	0.076
Cefdinir–Eudragit L100	0.135
SBA-15	NA
L-Arginine	0.260
L-Tryptophan	0.321
L-Phenylalanine	0.297

These results collectively indicate that both HSP and Flory–Huggins analyses predict strong miscibility of cefdinir with selected polymers and certain amino acids, whereas SBA-15 is likely to act primarily as a physical carrier rather than a solubility-enhancing excipient.

### Drug Content and Yield

Table 4 presents the production yield and drug content of the selected formulations (F1–F3). All formulations demonstrated high drug content with acceptable standard deviations, confirming uniform drug incorporation: 97.99% ± 2.75% for F1, 98.72% ± 2.92% for F2, and 99.33% ± 3.85% for F3. The highest production yield was observed for F3 at 84.47%, followed by F2 at 82.04%, while F1 showed the lowest yield of 78.56%.

**TABLE 4:** Results of yield and drug content of the selected formulations.

Formulation	Yield (%)	Drug content (%)
F1	78.56	97.986 ± 2.745
F2	82.04	98.11 ± 1.92
F3	84.473	99.330 ± 3.849

### Polarized Light Microscopy (PLM)

**FIGURE 1:**
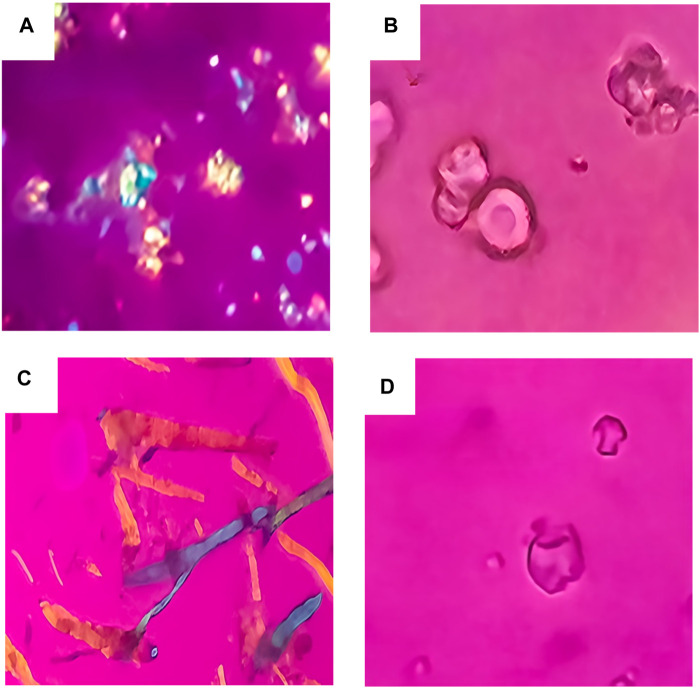
PLM images for: pure cefdinir **(A)**, F1 **(B)**, F2 **(C)** and F3 **(D)**.

PLM was employed as a preliminary screening tool to assess the solid-state form of cefdinir within the formulations. Pure cefdinir displayed characteristic acicular crystals with strong birefringence (Figure 1A). In contrast, the PVP K30-based solid dispersion (F1) showed no birefringence (Figure 1B), indicating successful amorphisation of the drug. Similarly, the co-amorphous formulation (F3) exhibited complete absence of birefringence (Figure 1D). Yet, no cefdinir crystals were detected in the SBA-15-based formulation (F2) (Figure 1C), implying molecular dispersion of the drug within the silica matrix.

### Thermal Stability via DSC

**FIGURE 2:**
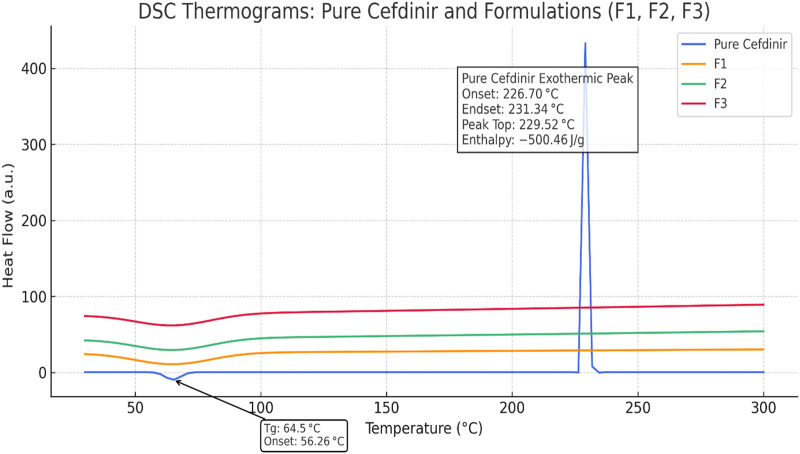
DSC thermograms of pure cefdinir, F1, F2, and F3 highlighting the exothermic activity.

The DSC thermogram of pure cefdinir (Figure 2) revealed a minor endothermic event around 65 °C corresponding to the glass transition temperature, followed by a sharp exothermic peak near 229.5 °C attributable to decomposition-associated melting. These observations align with known thermal behaviour of anhydrous cefdinir [38]. In contrast, none of the formulated systems (F1–F3) showed this high-temperature exothermic event, indicating suppression of thermal degradation through amorphisation and matrix stabilisation. While subtle baseline deviations were observed in the low-temperature region for the formulations, these transitions were broad and poorly defined, preventing reliable Tg determination under the applied experimental conditions.

### X-Ray Powder Diffraction

**FIGURE 3:**
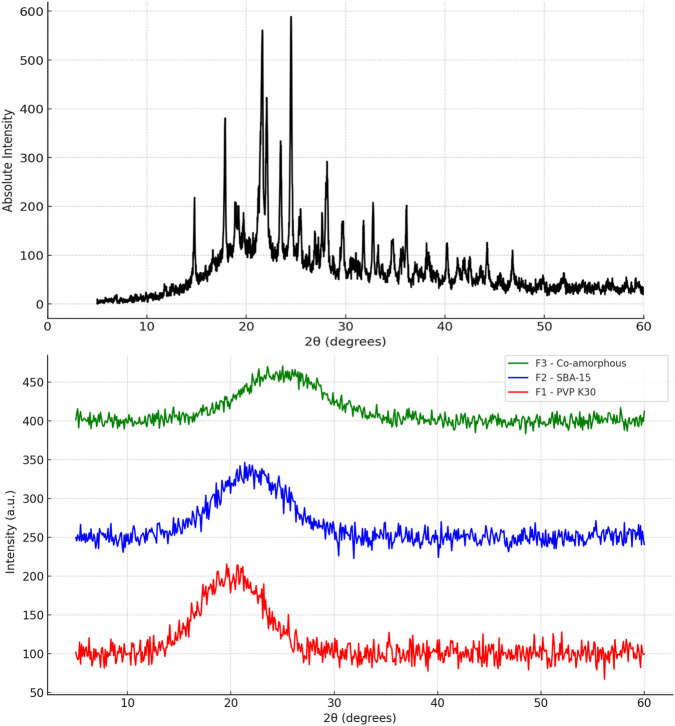
PXRD of: pure cefdinir, F1 (PVP-K30 based formulation), F2 (SBA-15-based), and F3 (Co-amorphous formulation) directly after preparation.

The PXRD pattern of pure cefdinir (Figure 3) exhibited sharp, intense peaks at 2θ values of 5.85°, 11.7°, 16.1°, 21.15°, 22.25°, 24.4°, 26.2°, and 28.8°, consistent with previous reports [39]. Conversely, the diffraction patterns for F1, F2, and F3 showed complete disappearance of sharp Bragg peaks, replaced by broad halos. This confirms the transformation of cefdinir into an amorphous state in all three formulations.

### Dissolution Studies

**FIGURE 4:**
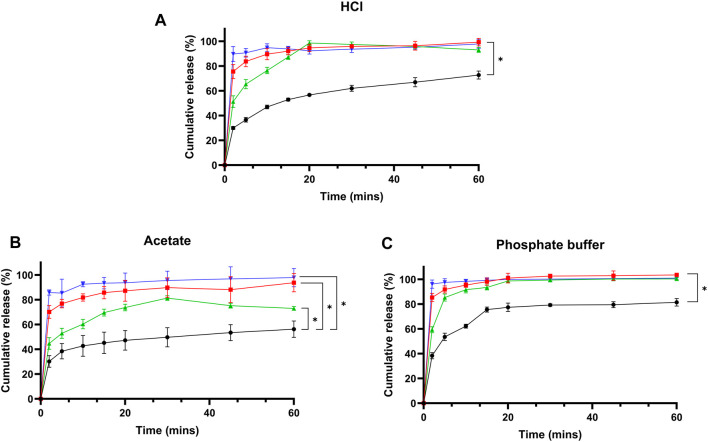
Drug release studies from pure cefdinir and the selected formulations in: **(A)** HCl buffer pH 1.2, **(B)** Acetate buffer pH 4.5, and **(C)** Phosphate buffer pH 6.8.

Dissolution profiles for formulations F1, F2, and F3 were evaluated in three media: HCl buffer (pH 1.2), acetate buffer (pH 4.5), and phosphate buffer (pH 6.8), as shown in Figures 4A–C respectively. In HCL, cefdinir showed the lowest release (72.72% ± 3.22%) after 60 min, while the release increased for F2 (92.95 ± 1.93) and F3 (97.81 ± 3.65) with the highest release achieved using F1 (99.33 ± 3.05). Yet, when the acetate buffer was used, F3 presented the highest release (97.81 ± 7.34) followed by 93.73% ± 7.1%, 72.99 ± 1.53, and 56.23 ± 6.6, for F1, F2, and pure cefdinir, respectively. However, when phosphate buffer was used, a similar trend to HCl medium was observed: F1 presented the highest release (103.45% ± 1.3%), while F2 and F3 showcased similar release results 100.34% ± 1.01% and 100.98 ± 1 for F2 and F3, respectively. Statistical analysis using Student’s t-test at each time point revealed that all formulations significantly improved drug release compared to pure cefdinir, corroborating our earlier studies [32, 33]. In both HCl and acetate buffers, formulations F1 and F3 outperformed F2, particularly during the initial three sampling intervals. However, no significant differences in dissolution were observed among the formulations in phosphate buffer.

### Stability Studies

#### Physical

**FIGURE 5:**
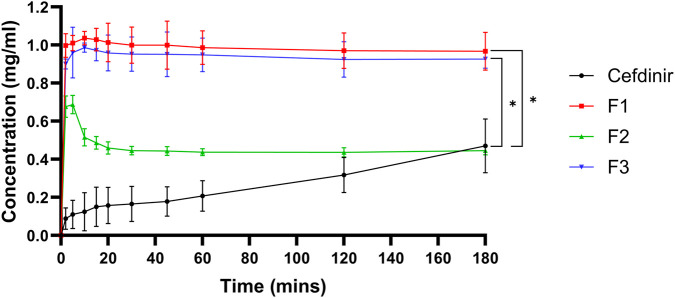
Results of physical stability in aqueous media.

Physical stability under non-sink aqueous conditions is presented in Figure 5. All formulations showed markedly higher solubility than crystalline cefdinir. Formulations F1 and F3 sustained drug concentrations at or near saturation throughout the testing period, demonstrating effective inhibition of recrystallisation. Although F2 initially showed a rapid increase in solubility, this was followed by a gradual decline, suggesting less effective maintenance of supersaturation.

**FIGURE 6:**
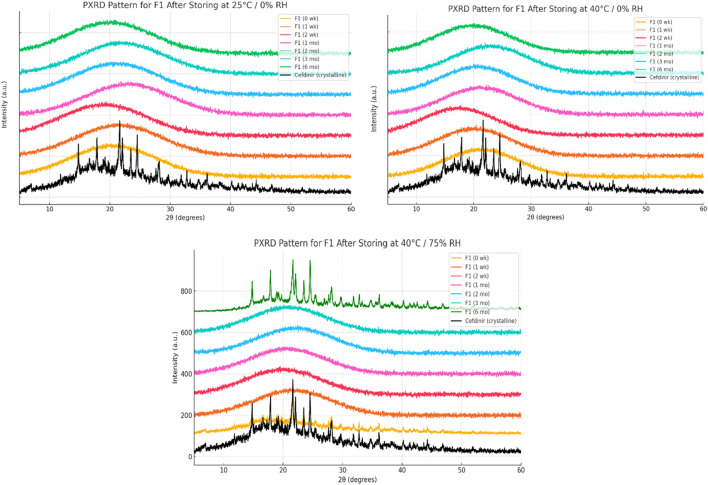
Physical stability results at solid state of formula F1.

**FIGURE 7:**
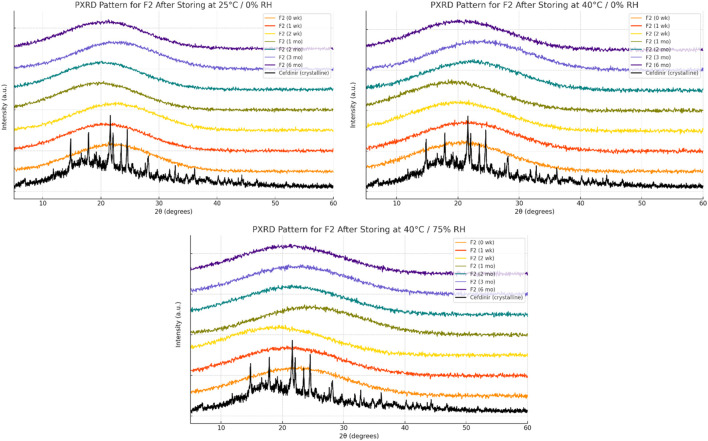
Physical stability results at solid state of formula F2.

**FIGURE 8:**
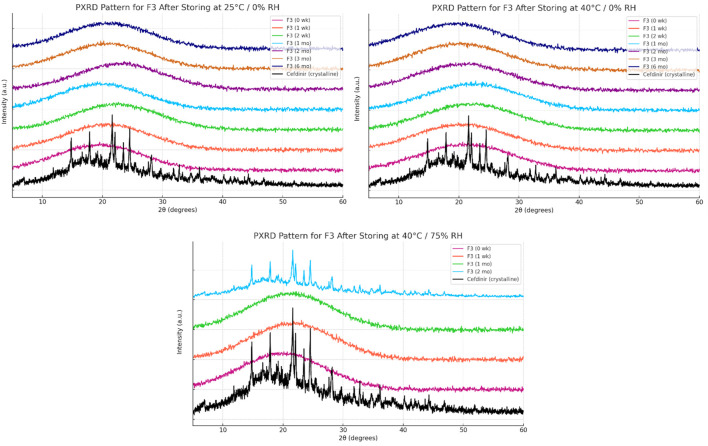
Physical stability results at solid state of formula F3.

Solid-state stability was further examined by PXRD after storage under various conditions (Figures 6–8). Under dry conditions (25 °C/0% RH and 40 °C/0% RH), both F1 and F3 remained amorphous with no evidence of recrystallisation. However, exposure to elevated humidity (40 °C/75% RH) led to partial recrystallisation in F1 and F3 after six and 2 months respectively. In contrast, F2 maintained its amorphous form throughout 6 months regardless of storage humidity, with PXRD patterns showing no crystalline peaks. These findings indicate superior physical stability for F2 in comparison to the other formulations.

#### Chemical

**FIGURE 9:**
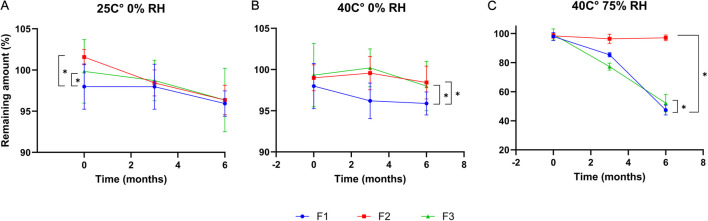
Chemical stability results after storing at: 25 °C/0%RH **(A)**, 40 °C/0% RH **(B)**, and 40 °C/75%RH **(C)**.

Chemical stability profiles over 6 months are illustrated in Figure 9. Under dry storage conditions (25 °C/0% RH and 40 °C/0% RH), all formulations retained more than 95% of cefdinir content, with no significant degradation detected. However, under accelerated humidity (40 °C/75% RH), significant drug degradation occurred (p < 0.05) in F1 and F3, with drug contents reduced to 47.39% ± 3.41% and 52.17% ± 6% respectively. In contrast, F2 retained 97.04% ± 1.97% of the drug with a higher significant amount (p < 0.05), highlighting its markedly enhanced chemical stability under humid conditions.

## Discussion

The compatibility of cefdinir with polymeric, mesoporous, and co-amorphous stabilisers was assessed using the Hansen Solubility Parameter (HSP) approach alongside the Flory–Huggins interaction parameter (χ) model. These theoretical frameworks serve as valuable preformulation tools, enabling the prediction of drug–excipient miscibility and guiding the selection of stabilisers for amorphous formulations. Based on the HSP analysis, low Ra values (<5 MPa^½^) indicated that PVP K30, HPMC 606, and all amino acids studied are highly compatible with cefdinir. This observation was further supported by Flory–Huggins χ values, which confirmed miscibility for all polymer-based systems (χ < 0.14). Among the polymers, HPMC 606 exhibited the lowest χ value (0.076), highlighting its potential as an effective stabiliser for amorphous cefdinir. Despite this, PVP K30 was selected for the polymer-based solid dispersion (F1), based on prior studies demonstrating its superior dissolution profile across multiple gastrointestinal-relevant media [32]. Moreover, the molecular weight-dependent solubility characteristics of PVP favour the formation of solid solutions, offering practical advantages for formulation development and processing [40].

The mesoporous silica SBA-15, in contrast, demonstrated poor solubility-based compatibility (high Ra), indicating that its primary role in formulations is likely as a physical carrier for controlled release rather than as a molecular stabiliser. The amino acids, particularly L-tryptophan and L-phenylalanine, are predicted to interact favourably with cefdinir, potentially serving as co-amorphous stabilisers or crystallisation inhibitors, although L-arginine’s high polarity may limit miscibility unless ionic interactions are exploited. Collectively, these findings underscore the utility of HSP and Flory–Huggins analyses in rational excipient selection, providing predictive guidance that complements experimental formulation studies. By integrating these theoretical models with prior dissolution and processing data, the selection of stabilisers such as PVP K30 can be optimised for developing stable, amorphous cefdinir formulations.

All three formulations achieved high drug content with acceptable standard deviations, confirming effective drug incorporation regardless of the stabilisation strategy employed (F1: 97.99% ± 2.75%; F2: 98.72% ± 2.92%; F3: 99.33% ± 3.85%). This indicates that both spray drying (used for F1 and F3) and solvent immersion (used for F2) produced uniform and consistent products. Regarding process yield, the co-amorphous system (F3) achieved the highest yield at 84.47%, likely due to enhanced powder recovery from improved flowability and reduced stickiness of amino acid-based matrices during spray drying. SBA-15 (F2) demonstrated a high yield of 82.04%, confirming the viability of the solvent immersion method despite a comparatively low drug loading of 37.18%. The PVP K30-based solid dispersion (F1) showed the lowest yield at 78.56%, probably owing to product adherence within the spray dryer chamber or collection losses commonly associated with hydrophilic polymers. Taken together, these findings indicate all systems effectively incorporate the drug, with F3 offering the best process recovery and F2 providing an excellent balance between yield, drug content, and defined loading, making both promising candidates for further development. While saturated solubility studies were not explicitly conducted, the dissolution media volumes were deliberately chosen to exceed the expected maximum solubility of cefdinir, and aliquot replacement ensured that drug concentrations remained well below saturation throughout testing. The complete dissolution of all formulations across the testing period indicates that sink conditions were effectively maintained, supporting reliable interpretation of dissolution kinetics. Although direct solubility measurements could provide additional confirmation, the current approach provides a robust and practical assessment of formulation performance.

Polarised light microscopy (PLM) further confirmed successful amorphisation of cefdinir in all three formulations. The PVP-based solid dispersion (F1) exhibited no birefringence, as expected from typical amorphous solid dispersions [36, 41, 42]. Although SBA-15 (F2) is structurally amorphous, it displayed birefringence under PLM due to the hexagonally ordered mesoporous structure, which can cause anisotropic light transmission mimicking birefringence [36, 43, 44]. Importantly, no visible cefdinir crystals were detected in the F2 images, suggesting effective confinement of the drug in an amorphous state within the mesoporous matrix. The co-amorphous formulation (F3) showed zero birefringence, indicating the formation of a homogeneous amorphous blend with the amino acid coformers. These observations align well with the theoretical predictions from the Hansen and Flory–Huggins models, which suggest strong miscibility between cefdinir and both polymeric and amino acid-based carriers. Thus, PLM confirmed the successful amorphisation of cefdinir across all three systems, supporting their further evaluation in stability and dissolution studies.

Thermal analysis by DSC was primarily employed to evaluate the thermal behaviour and degradation profile of cefdinir in its crystalline form compared with the processed formulations, rather than to resolve subtle glass transition events. Pure crystalline cefdinir exhibited a sharp exothermic peak at approximately 229.5 °C (Figure 2), which is attributed to thermal decomposition occurring during melting, likely initiated by nucleophilic attack on the β-lactam ring, as reported previously for cephalosporins [45]. This degradation-associated thermal event reflects the inherent thermal instability of crystalline cefdinir. In contrast, none of the formulated systems (F1–F3) displayed this exothermic peak across the scanned temperature range, indicating suppression of thermally induced degradation following formulation. In the PVP K30-based solid dispersion (F1), drug–polymer hydrogen bonding and reduced molecular mobility are likely responsible for inhibiting degradation pathways. For the SBA-15 formulation (F2), nanoscale confinement within mesoporous channels physically restricts molecular rearrangement and limits thermal decomposition. In the co-amorphous system (F3), stabilisation is attributed to hydrogen bonding and ionic interactions between cefdinir and the amino acid coformers, which further reduce molecular mobility during heating. These observations are consistent with previous reports demonstrating enhanced thermal stability of drugs formulated as polymeric solid dispersions, mesoporous silica systems, and co-amorphous mixtures [46–51].

Although minor baseline deviations were observed for the formulations at lower temperatures, these events were broad and poorly resolved and could not be reliably assigned to distinct glass transition temperatures (Tg). This behaviour is typical of multicomponent amorphous systems, where overlapping relaxation processes, compositional heterogeneity, and strong drug–excipient interactions often obscure discrete Tg signals, particularly under conventional DSC conditions. As such, the absence of clearly defined Tg transitions does not contradict the amorphous nature of the formulations.

To confirm the solid-state form suggested by DSC, X-ray powder diffraction (XRPD) analysis was conducted subsequently on pure cefdinir and all processed formulations. XRPD results demonstrated the complete absence of characteristic crystalline cefdinir diffraction peaks in all formulations (Figure 3), confirming successful amorphisation and corroborating the DSC findings. Taken together, DSC and XRPD provide complementary evidence that formulation effectively stabilised cefdinir by eliminating crystalline melting and degradation behaviour while maintaining the drug in an amorphous state. X-ray powder diffraction (PXRD) analysis reinforced these findings, showing the absence of sharp diffraction peaks characteristic of crystalline cefdinir in all formulations (Figure 3). This complements the PLM results and confirms the effective conversion of cefdinir into its amorphous form at the time of preparation.

The enhanced dissolution profiles observed for all three formulations compared to pure cefdinir are primarily attributed to amorphisation. The amorphous form possesses higher molecular mobility and internal energy, leading to increased apparent solubility and faster dissolution rates [16, 17]. These solid-state changes are crucial in overcoming the poor solubility inherent to crystalline cefdinir. Additionally, chemical modifications in formulations F1 and F3 further contributed to improved dissolution performance: F1 formed a sodium salt that increased ionic character at low pH, while F3’s arginine salt enhanced solubility via ionisation. Together, these solid-state and chemical alterations effectively address cefdinir’s intrinsic solubility limitations.

The improved physical stability in aqueous media observed for F1 and F3 is likely due to their ability to inhibit recrystallisation and maintain cefdinir in solution. PVP K30 and amino acids such as arginine and phenylalanine increase cefdinir’s solubility and prevent crystallisation caused by supersaturation through kinetic stabilisation and salt formation [50, 52, 53]. Conversely, SBA-15 (F2) failed to maintain amorphous stability in solution, evidenced by a decrease in drug concentration after 10 min. This instability may arise from rapid drug release causing transient supersaturation and subsequent recrystallisation. Water penetrating the silica structure might expel the drug, leading to precipitation. Hence, while mesoporous silica facilitates rapid dissolution, it does not adequately suppress recrystallisation under non-sink aqueous conditions unless combined with nucleation or crystal growth inhibitors.

The solid-state amorphous stability of the formulations was influenced by both the stabilising matrix and storage conditions. F1 and F3 maintained amorphous stability for extended periods under dry conditions, likely due to polymer viscosity and ionic interactions restricting molecular mobility [54, 55]. Increases in glass transition temperature (Tg) and strong excipient–drug interactions outweighed any crystallisation tendencies [18]. However, neither formulation resisted recrystallisation at 75% relative humidity (RH), probably because moisture absorption by hygroscopic PVP K30 and amino acids reduced Tg and induced phase separation [56]. In contrast, F2 performed best under both dry and humid conditions. The mesoporous structure of SBA-15 physically limited drug mobility through nanoconfinement and possibly formed chemical bonds with silanol groups on the silica surface, as the negatively charged silica surface favours forming hydrogen bonds with molecules [57–59]. PXRD analysis showed no recrystallisation for 6 months, even below 75% RH, demonstrating excellent silica-mediated stability.

Chemical stability studies showed all three formulations remained stable for 6 months under dry storage, with drug content decreasing by less than 5%, meeting ICH guidelines [20]. However, under accelerated humid conditions (40 °C/75% RH), F1 and F3 underwent significant degradation (P < 0.05), likely due to moisture sorption by hygroscopic components (PVP K30 and amino acids), which facilitated degradation. In contrast, the SBA-15-based formulation (F2) remained highly stable even in humid conditions, presumably because drug confinement within silica pores minimised exposure to moisture and physical isolation from degradation initiators. This aligns with previous reports indicating that silica carriers enhance chemical stability by restricting molecular mobility and protecting against hydrolysis [60–62] The superior stability of F2 not only ensures consistent drug release but also supports its potential for targeted oral delivery, as the drug remains protected until it reaches the intended site of action. Furtehrmore, mesoporous-based systems have been reported for targeting cellular organelles such as the mitochondria [63], which gives them an additional benefit for targeting applications. Therefore, F2 is the most chemically stable formulation, while F1 and F3 require protection from moisture.

## Conclusion

This study presents a detailed comparison of three amorphisation strategies to enhance the dissolution and stability of cefdinir, a poorly water-soluble antibiotic: polymer-based solid dispersion (PVP K30), mesoporous silica (SBA-15), and co-amorphous formulations with L-arginine and L-phenylalanine. Hansen solubility parameters and Flory–Huggins interaction theory were used to predict drug–carrier compatibility and guide stabiliser selection. Experimental results confirmed complete amorphisation in all formulations, with polymeric and co-amorphous systems demonstrating superior dissolution compared to the mesoporous silica-based formulation. Stability studies showed that amorphisation enhanced cefdinir’s thermal stability and slowed recrystallisation. However, long-term stability testing under revealed clear differences: the mesoporous silica system exhibited excellent chemical and physical stability, even in humid environments, due to molecular confinement within the silica matrix. Conversely, the polymer and co-amorphous formulations were less stable under humid conditions, likely due to the hygroscopic nature of their stabilisers. These findings highlight the importance of choosing formulation strategies according to the required stability profile and storage conditions. The enhanced stability of the silica-based formulation also supports its potential for targeted delivery, ensuring the drug remains protected until reaching intended site. The study further reinforces the valuable role of theoretical modelling in the rational design of amorphous drug formulations.

## Summary Table

### What Is Known About This Subject

- Amorphisation improves drug solubility and dissolution but requires careful stabiliser selection.
- Polymeric, mesoporous silica, and co-amorphous systems are common approaches to stabilise drugs.
- Theoretical models help predict drug–excipient compatibility to optimise formulation design.

### What This Paper Adds

- Theoretical modelling effectively guided selection of compatible stabilisers, confirmed by experiments.
- All three strategies fully amorphised cefdinir, with polymer and co-amorphous systems enhancing dissolution.
- Mesoporous silica offered superior chemical and physical stability under humid conditions despite slower release.

## Concluding Statement

This work represents an advance in biomedical science by demonstrating how rational selection of amorphisation strategies, guided by theoretical modelling, can optimise drug dissolution and stability, ultimately enhancing formulation design for poorly soluble medicines.

## Data Availability

The original contributions presented in the study are included in the article/supplementary material, further inquiries can be directed to the corresponding authors.
